# Prognostic Role of the Expression of Latent-Membrane Protein 1 of Epstein–Barr Virus in Classical Hodgkin Lymphoma

**DOI:** 10.3390/v13122523

**Published:** 2021-12-15

**Authors:** Antonio Santisteban-Espejo, Jose Perez-Requena, Lidia Atienza-Cuevas, Julia Moran-Sanchez, Maria del Carmen Fernandez-Valle, Irene Bernal-Florindo, Raquel Romero-Garcia, Marcial Garcia-Rojo

**Affiliations:** 1 Department of Pathology, Puerta del Mar University Hospital, 11009 Cadiz, Spain; jose.perez.sspa@juntadeandalucia.es (J.P.-R.); lidia.atienza.sspa@juntadeandalucia.es (L.A.-C.); marcial.garcia.sspa@juntadeandalucia.es (M.G.-R.); 2 Institute of Research and Innovation in Biomedical Sciences of the Province of Cadiz (INiBICA), 11009 Cadiz, Spain; iriberni@hotmail.com (I.B.-F.); raquel.romero.garcia@juntadeandalucia.es (R.R.-G.); 3 Department of Medicine, Faculty of Medicine, University of Cadiz, 11003 Cadiz, Spain; juliamorsan@gmail.com; 4 Department of Hematology and Hemotherapy, Puerta del Mar University Hospital, 11009 Cadiz, Spain; macafeva@hotmail.com

**Keywords:** B-cell lymphomas, classical Hodgkin lymphoma, Epstein–Barr virus, Latent-Membrane Protein 1, risk-adjusted therapy

## Abstract

The prognostic impact of the presence of Epstein–Barr virus (EBV) in classical Hodgkin lymphoma (cHL) is controversial. Previous studies reported heterogeneous results, rendering difficult the clinical validation of EBV as a prognostic biomarker in this lymphoma. The objective of this study was to evaluate the survival impact of the expression of EBV Latent-Membrane Protein 1 (EBV-LMP1) in tumoral Hodgkin–Reed–Sternberg (HRS) cells of primary diagnostic samples of cHL. Formalin-Fixed Paraffin-Embedded (FFPE) lymph node samples from 88 patients with cHL were analyzed. Patients were treated with the standard first-line chemotherapy (CT) with Adriamycin, Bleomycin, Vinblastine and Dacarbazine (ABVD) followed by radiotherapy. The Kaplan–Meier method and the Cox proportional hazards model were used for carrying out the survival analysis. In order to investigate whether the influence of EBV was age-dependent, analyses were performed both for patients of all ages and for age-stratified subgroups. In bivariate analysis, the expression of EBV was associated with older age (*p =* 0.011), mixed cellularity subtype cHL (*p* < 0.001) and high risk International Prognostic Score (IPS) (*p =* 0.023). Overall survival (OS) and progression-free survival (PFS) were associated with the presence of bulky disease (*p =* 0.009) and advanced disease at diagnosis (*p* = 0.016). EBV-positive cases did not present a significantly lower OS and PFS in comparison with EBV-negative cases, for all ages and when stratifying for age. When adjusted for covariates, absence of bulky disease at diagnosis (HR: 0.102, 95% CI: 0.02–0.48, *p =* 0.004) and limited disease stages (I–II) (HR: 0.074, 95% CI: 0.01–0.47, *p =* 0.006) were associated with a significant better OS. For PFS, limited-disease stages also retained prognostic impact in the multivariate Cox regression (HR: 0.145, 95% CI: 0.04–0.57, *p =* 0.006). These results are of importance as the early identification of prognostic biomarkers in cHL is critical for guiding and personalizing therapeutic decisions. The prognostic role of EBV in cHL could be modulated by the type of CT protocol employed and interact with the rest of presenting features.

## 1. Introduction

Classical Hodgkin lymphoma (cHL) is a B-cell neoplasm characterized by scarce neoplastic Hodgkin’s Reed–Sternberg (HRS) cells surrounded by a heterogeneous tumoral microenvironment composed of B and T lymphocytes, eosinophils, macrophages, plasmatic cells, neutrophils and histiocytes [1]. The integration of the genome of the Epstein–Barr virus (EBV) in HRS cells has been demonstrated in a variable proportion of cases, ranging from 20% in industrialized countries to 90% in less developed countries [2]. Moreover, individuals with a history of infectious mononucleosis have an approximately three-fold increased risk of developing cHL [3]. Because EBV is clonal in HRS cells, this implicates its direct role in the oncogenic process [4]. In EBV-positive cHL, latent infection is considered a causal event [5].

Despite the fact that the detection of EBV in Formalin-Fixed Paraffin-Embedded (FFPE) tissue has been consistently associated with older age, male sex and mixed cellularity subtype, its prognostic significance in cHL is controversial. Some studies have identified a shorter survival in EBV-positive cases, especially in older adults [6], but a protective influence [7,8,9] and a lack of survival impact [10,11] were also reported. The heterogeneity of these results could be attributed to differences in sample sizes, follow-up times, methods to identify the presence of EBV, statistical analysis and clinical endpoints defined to evaluate prognostic relevance. 

Notably, an important component of variability when assessing the association of EBV with survival rates in this type of cancer could be due to the different treatment protocols included in the studies: radiotherapy alone, MOPP (Mechlorethamine, Vincristine, Procarbazine, Prednisone) and ABVD (Adriamycin, Bleomycin, Vinblastine, Dacarbazine) with or without radiotherapy [7]; ABVD and variants with or without radiotherapy [8]; and MOPP/ABV (Adriamycin, Bleomycin, Vinblastine), EBVP (Epirubicin, Bleomycin, Vinblastine, Prednisone), or MOPP and LOPP (Lomustine, Vincristine, Procarbazine, Prednisone) [6]. In some studies reporting a statistically significant association between EBV infection and survival outcomes, treatment modalities [12] or types of chemotherapy regimens considered [9] are not always clearly indicated, rendering difficult the clinical interpretation of the survival analysis. 

The present study investigates the prognostic impact of EBV in a cohort of patients with cHL diagnosed at the Puerta del Mar University Hospital in Cadiz, Spain. All patients received the same first-line chemotherapy schedule with the ABVD regimen as it constitutes the current standard first-line protocol in cHL and to avoid bias due to the type of treatment received. Additionally, to investigate whether the influence of EBV latent infection was age-dependent, overall survival (OS) and progression-free survival (FFS) rates were calculated in all patients and age-stratified subgroups. 

## 2. Materials and Methods

### 2.1. Patients and Samples

Formalin-Fixed Paraffin-Embedded (FFPE) lymph node specimens from 88 patients diagnosed with cHL at the Pathology Department of the Puerta del Mar University Hospital between the years 2009 and 2020 were selected for the study. From an initial sample of 167 cases, we selected 88 cases for final statistical analysis that satisfied all the following criteria: primary diagnosis of cHL, patients candidates for intensive treatment, first-line chemotherapy with the ABVD regimen with or without the addition of radiotherapy and immunohistochemical evaluation of EBV-Latent-Membrane Protein 1 (EBV-LMP1) in the diagnostic sample. All selected cases were evaluated by the same pathologists (JPR and LAC) to ensure homogeneity in the results. Although the presence of EBV was previously reported in cases of nodular lymphocyte-predominant Hodgkin lymphoma (NLPHL) [13,14], we did not include cases of this entity because of its less frequent association with EBV infection in comparison with cHL. The diagnoses were made by experienced hematopathologists in accordance with the 2016 World Health Organization criteria for the diagnosis of lymphoid neoplasms [15]. The collection of clinical data was performed from the electronic health record of the patients. We evaluated seven clinicopathological variables in each case: sex, age, histological subtype, Ann Arbor stage with Cotswold’s modifications, presence of B symptoms at diagnosis (fever, drenching night sweats and loss of more than 10 percent of body weight over 6 months prior to diagnosis), bulky disease (mass in the chest that is one-third the width of the chest, or any lymph node mass greater than 10 cm) and EBV status (immunohistochemistry for EBV-LMP1 positive or negative in HRS cells). Two prognostic variables were recorded: the International Prognostic Score (IPS), categorized as low risk (0–2) and high risk (3–7) [16], and the German Hodgkin Study Group, Cologne, Germany (GHSG) score, categorized as limited, intermediate and advanced stages [17]. One variable of response to therapy, measured as the percentage of patients with primary refractory or relapsed disease (R/R patients), was also evaluated. All the samples and data were collected following the technical and ethical procedures of the local institutions and in accordance with the Helsinki Declaration. The study was approved by the local Ethics Committee (protocol code 1167-N-21). 

### 2.2. Epstein–Barr Virus Detection

An immunohistochemical study to detect the expression of EBV in tumoral HRS cells was performed using anti-human mouse Epstein–Barr Virus/LMP1 Monoclonal Antibody (Clone CS1-4) (Master Diagnostica, Inc., Granada, Spain). We used EBV-LMP1 as a marker of EBV presence. Previous studies showed a very good interobserver agreement between LMP1 and EBV-encoded early RNAs (EBERs) in situ hybridization to identify an EBV-positive cHL [7,18,19]. Formalin-fixed 4 µm sections from paraffin-embedded blocks were deparaffinized and rehydrated and subsequently pretreated by heat for 20 min at 95 °C for antigen retrieval. The immunohistochemistry automated staining was performed using BenchMark ULTRA IHC/ISH System (Ventana Medical Systems Inc., Tucson, AZ, USA), following the manufacturer’s protocols. EBV-LMP1 expression was manually evaluated by two different pathologists in each case. It was considered positive if any HRS cell was stained. Known EBV-positive cases of cHL were used as positive controls. 

### 2.3. Statistical Analysis

Descriptive statistics were performed for the ten variables evaluated in each case. For statistical inference, the χ^2^ test and the Fisher exact test were used for the comparison between categorical variables. Only cases with all of the required data were selected for the final statistical analysis. Therefore, the analysis was performed in 88 patients from the initial cohort of 167 patients. In order to evaluate the clinical impact of the variables collected, univariate and multivariate survival analyses were performed. In the univariate analysis, the Kaplan–Meier method and the log-rank test were used to compare the survival distributions of patients [20]. This analysis was performed for patients between 19 and 49 years old, ≥50 years old and for all ages. The Cox proportional hazards model was used in the multivariate analysis to evaluate the influence of the different covariates in survival [21]. Ann Arbor stages I and II and limited and intermediate stages of the GHSG score could not be separated because no patients died neither with stage I nor with limited stages; therefore, stages I–II and limited-intermediate stages were analyzed as joints groups. All *p* values were two-sided, and a level of probability below 0.05 was considered significant. The IBM SPSS software (version 15.0) (SPSS Inc., Chicago, IL, USA) was used to perform the statistical analysis. 

### 2.4. Outcome

The OS and the PFS were used as clinical endpoints for the univariate and multivariate survival analysis. The OS was defined as the time interval between the initial histopathological diagnosis and death, lost to follow-up or end of the study. The PFS was defined as the time interval between the initial histopathological diagnosis and the first progression or relapse after achieving a complete remission (CR). Data were collected until June 2021. The evaluation of the response to the treatment was performed by positron emission tomography/computed tomography ((18)F-FDG PET/CT) following the revised Cheson criteria [22]. 

## 3. Results

### 3.1. Patient Demographics

**Figure 1:**
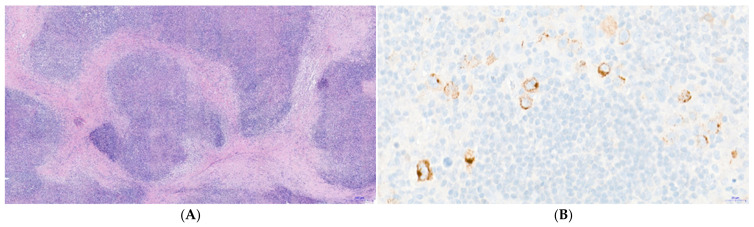
Histopathology of classical Hodgkin lymphoma (cHL) and expression of Epstein–Barr virus Latent-Membrane Protein 1 (EBV-LMP1). (**A**) Hematoxylin-Eosin (H&E) staining showing fibrotic bands that deimitate a nodular pattern in a case of nodular sclerosis cHL (magnification 4×) (**B**) Immunohistochemistry for Epstein–Barr Virus Latent Membrane Protein 1 (EBV-LMP1) shows a cytoplasmic and membranous staining in tumoral Hodgkin and Reed–Sternberg (HRS) cells (magnification 20×). Positivity for LMP1 is restricted to cells that are cytologically identifiable as part of the tumor clone, i.e., HRS cells. Cells in the inflammatory background (peritumor microenvironment) are shown as negative for LMP1 expression, following the established recommendations for the interpretation of this marker [19].

Table 1 shows the description of the characteristics of the patients included in the study. A total of 56% of the patients were male, and most of them (68%) were 30 years old or older. The mean age at diagnosis was 39 years (range: 19–82). Nodular sclerosis cHL was the most frequent histological subtype (63%), followed by mixed cellularity (18%). Representative histopathology of a case of nodular sclerosis cHL is shown in Figure 1A. Patients reported B symptoms in 60% of the cases, and a minority (14%) presented with bulky disease. The expression of EBV-LMP1 was detected in 41% of the samples studied. Figure 1B shows an exemplary pattern of positive immunostaining for EBV-LMP1 in a case of mixed cellularity subtype cHL. Positivity for LMP1 was widespread (>50%) among HRS cells in the cHL samples defined as EBV-positive. 

As recommended in published Guidelines for interpreting EBER in situ hybridization and LMP1 immunohistochemical tests for detecting EBV in cHL [19], the positivity for LMP1 in Figure 1B is restricted to the tumoral clone. Only the larger and multinucleated cells that correspond cytologically to HRS cells showed immunohistochemical positivity for LMP1. Furthermore, the cellular background (lymphocytes, eosinophils, granulocytes, histyocites) was negative for LMP1, confirming that EBV expression was restricted to the tumoral population. 

Regarding the prognostic scores, the IPS was between 3 and 7 points in the 47% of the cases and, according to the GHSG score, most of the patients presented with advanced stages at diagnosis (74%), followed by intermediate stages (18%) and limited stages (8%). Regarding the response to first-line treatment, most of the patients achieved a CR with te first-line therapy (77%). 

Table 2 shows the comparison between all the variables adjusted for the EBV infection status. A statistically significant association was obtained for the variables age, histological subtype and the IPS. Among the EBV-positive cases, 30/36 (83%) were 30 years old or older (*p =* 0.011). The mixed cellularity subtype was the most frequent histology associated with EBV with 15/36 (42%) of EBV-positive cases of cHL (*p* < 0.001). Finally, a high-risk IPS was also more prevalent in the EBV-positive cHL with 22/36 cases (61%) with an IPS above three points (*p =* 0.023).

### 3.2. Survival Analysis

#### 3.2.1. Univariate Analysis

Table 3 and Table 4 show the results of the Kaplan–Meier analysis for the OS and PFS, respectively. The presence of bulky disease at diagnosis was statistically significantly associated with a different distribution in the OS curves (*p =* 0.009). Patients with the voluminous disease had shorter survival (mean OS: 52 months; 95% CI: 37–67 months) in comparison with patients without the bulky disease (mean OS: 93 months; 95% CI: 82–104 months).

The Ann Arbor stage was also statistically associated with a difference in PFS rates (*p* = 0.016). Patients with stage III (mean PFS: 73 months; 95% CI: 35–111 months) and stage IV (mean PFS: 76 months; 95% CI: 61–91 months) had a shorter PFS in comparison with stages I–II (mean PFS: 89 months: 95% CI: 82–97 months). 

The EBV infection status was not statistically associated neither with OS (*p =* 0.642) nor with PFS (*p =* 0.856). However, OS and PFS were both higher in patients testing positive for EBV-LMP1 in diagnostic samples. The mean OS for the EBV-LMP1 negative cases and EBV-LMP1 positive cases were 87 months (95% CI: 75–99 months) and 93 months (95% CI: 78–108 months), respectively. The mean PFS for the EVB-LMP1 negative cases and EBV-LMP1 positive cases were 86 months (95% CI: 74–97 months) and 91 months (95% CI: 71–111 months), respectively.

#### 3.2.2. Analysis by Age Subgroups

**Figure 2:**
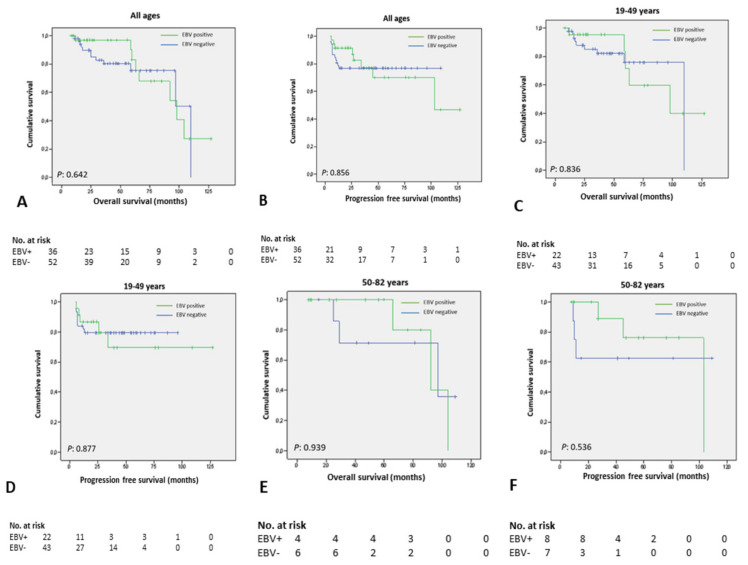
Survival impact of EBV adjusted for age-stratified subgroups. (**A**,**B**) Overall survival and progression-free survival for all ages; (**C**,**D**) Overall survival and progression-free survival for patients between 19 and 49 years old; (**E**,**F**) Overall survival and progression-free survival for patients above 50 years old.

To assess whether the prognostic impact of EBV was age-dependent, we also performed survival analysis according to age-stratified subgroups. No statistically significant differences were observed for patients between 19 and 49 years, 50 and 82 years and for all ages (Figure 2). However, survival times tended to be more favorable in patients testing positive for EBV-LMP1 when adjusted for age. 

In the group aged 19–49 years old, the mean OS of EBV-LMP1 positive cases and EBV-LMP1 negative cases were 92 months (95% CI: 71–114 months) and 91 months (95% CI: 78–103 months). For the same age range, the mean PFS of EBV-LMP1 positive cases and EBV-LMP1 negative cases were 95 months (95% CI: 70–120 months) and 78 months (95% CI: 67–88 months), respectively.

In patients above 50 years, the mean OS of EBV-LMP1 positive cases and EBV-LMP1 negative cases were 92 months (95% CI: 76–108 months) and 81 months (95% CI: 55–107 months). In this age subgroup, the mean PFS was 87 months for EBV-LMP1 positive cases (95% CI: 64–111 months) and 72 months (95% CI: 39–105 months) for EBV-LMP1 negative cases.

### 3.3. Multivariate Analysis

The Cox proportional hazards model for OS and PFS had a good fit (OS: χ^2^ = 20.664; *p* = 0.024. PFS: χ^2^ = 19.999; *p* = 0.029). Table 5 shows the results of the multivariate survival analysis. The variables sex, Ann Arbor stage and bulky disease were independently associated with OS in the multivariate analysis. Female patients had a hazard ratio (HR) of 4.121 (95% CI: 1.11–15.27, *p =* 0.034) considering male patients. Patients with stages I–II had a HR of 0.074 (95% CI: 0.01–0.47, *p =* 0.006) considering patients with stage IV. Patients without bulky disease at diagnosis had a HR of 0.102 (95% CI: 0.02–0.48, *p =* 0.004) considering patients with bulky disease. Expression of EBV-LMP1 was not statistically associated with OS in the multivariate model (*p =* 0.370). 

The only presenting feature associated with PFS in the Cox regression model was the Ann Arbor stage. Patients with limited stages (I–II) had a HR of 0.145 (95% CI: 0.04–0.57, *p =* 0.006) considering patients with stage IV. Expression of EBV-LMP1 was not statistically associated with PFS in the multivariate Cox regression (*p =* 0.588).

## 4. Discussion

The identification of prognostic factors in cHL is essential for adjusting therapy to individual risk. One of these parameters, available at initial routine diagnosis, is the presence of EBV in neoplastic HRS cells. It is widely accepted that EBV plays a role in lymphomagenesis and, as demonstrated for other tumors such as nasopharyngeal carcinoma [23] and the endemic variant of Burkitt’s lymphoma [24], the genome of EBV is clonal in HRS cells.

The integration of the EBV genome in the tumoral cell population of cHL was originally described in 1985 by Poppema S et al. [4]. Subsequent studies aimed to elucidate the molecular pathways activated by EBV in HRS cells. Four major hallmarks of EBV-related oncogenesis in cHL have been described: (1) EBV oncogenic proteins (essentially, LMP1 and LMP2A) can rescue germinal center (GC) B cells with crippled mutations from apoptosis [25]; (2) EBV enhances growth, proliferation and transformation of naïve B cells by upregulating the nuclear factor kappa beta (NFKB) pathway, which is constitutively activated in virtually all cHL derived cell lines [26]; (3) three different gene expression programs (growth program or latency III, default program or latency II and latency program or latency 0) are differentially expressed during EBV life cycle, being the type II latency the main genetic program expressed in HRS cells [27]; and (4) although traditionally their contribution to EBV-related oncogenesis has received less attention, an increasing amount of data support the role of transcriptional factors responsible for the abortive lytic cycle of EBV (i.e., ZEBRA) in the development of EBV-driven malignancies [28].

Regarding this, a previous study showed that a statistically significant increase exists in the levels of soluble ZEBRA protein (sZEBRA) in serum samples of patients with post-transplant lymphoproliferative disease (PTLD) in comparison with EBV-seronegative subjects and immunocompetent individuals with EBV serological reactivation [29]. To the best of our knowledge, there is no literature aiming to evaluate the levels of sZEBRA during the course of EBV-positive cHL patients, but considering the limited amount of data regarding the relevant role of ZEBRA for EBV-driven oncogenesis, future research in this area is expected.

Regarding this, recent studies also showed that lymphoid neoplasms are more frequent in lymphoblastoid cell lines (LCLs) derived with wild-type EBV than in LCLs derived with BZLF1-KO-EBV, the gene coding ZEBRA [30]. Furthermore, ZEBRA actions are not restricted to the infected cell because they can potentially penetrate any cell through its cell-penetrating domain [31]. This capability is of particular interest in a neoplam such as cHL, in which the reshaping, organization and dynamic interactions between HRS cells and the different cell types integrating the tumor microenvironment could influence the response to therapy.

The results presented in this study are in accordance with previous works reporting an association between the expression of EBV in cHL, older age and mixed cellularity subtype [2,32]. However, the reasons for these observations are not yet fully understood. Particularly, immunosenescence seems to play an important role in the higher prevalence of EBV in the elderly, as the impaired immune system could not respond efficiently to viral infection, leading to EBV reactivation and oncogenic transformation [12]. Additionally, LMP1 has proven to act as a member of the tumoral necrosis factor (TNF) family receptors, thus, contributing to the constitutive activation of the NFKB signaling pathway, which is known to support the proliferation of HRS cells and contribute to immune evasion [33,34]. Particularly, a type II latent program was shown to be expressed in HRS cells of cHL [27]. In this state, HRS cells turn off the expression of all latency proteins except EBNA1, LMP1 and LMP2. This fact is of relevance because HRS cells characteristically show rearrangement of genes coding the heavy and light chains of the immunoglobulins but without expressing the B cell receptor (BCR) on the cell surface. The expression of the BCR constitutes a prerogative for B-cell surveillance and evasion of apoptosis during somatic hypermutation occurring in the GC. Consequently, an essential function of latency type II proteins of EBV is to promote the evasion of apoptosis in GC. As a consequence, EBV could confer a proliferative advantage to the tumoral clone and promote its surveillance [25].

Furthermore, LMP1 lytic isoforms act as a CD40 receptor on the cell surface of HRS cells, thus, mimicking the functional activation of the TNFα receptor and promoting the evasion of apoptosis and cell proliferation, which is a key event in the pathogenesis of cHL [35].

Nevertheless, despite the significant knowledge about the biological role of EBV in cHL, its prognostic significance is not well established. There is contradictory literature, ranging from studies showing a favorable, negative, or lack of association between the expression of EBV and the clinical outcomes of patients with cHL. Methodologies vary importantly in terms of sample sizes, power of statistical techniques employed, clinical endpoints defined, therapeutic regimens considered and techniques used to detect the presence of EBV.

Previous studies attempted to compare existing tests for the evaluation of EBV in cHL. A study [36] analyzed 59 cases of cHL in FFPE samples to compare the sensitivities of LMP1, EBER in situ hybridization and polymerase chain reaction (PCR) for EBV detection. The presence of EBV was detected in 39/59 (66.1%) and 40/59 (67.8%) cases with LMP1 and EBER, respectively. The PCR showed the highest detection rate (44/59; 74.6%). However, PCR is not recommended to routinely evaluate the expression of EBV in cHL because it does not allow the visual identification of infected cells, and previous studies have also shown that in a proportion of PCR-positive cases of cHL, EBV was localized in “bystander” lymphocytes that do not constitute part of the tumoral clone [37]. A limitation of our study is that correlations with EBER were not performed, and this would strengthen the findings and prognostic associations obtained.

Some studies showed a positive prognostic effect of the expression of EBV-LMP1 in the response to therapy of patients with cHL. A study from a Spanish group showed the independent significance of the expression of EBV-LMP1 as a favorable prognostic marker in relation to OS [7]. Lately, the same group showed a larger OS, failure-free survival (FFS) and better complete remission rates in EBV positive cases when excluding patients treated only with radiotherapy [8]. The selection of cases in these studies, however, is based on the Rye classification, and this could introduce a proportion of NLPHL cases, which constitutes an entity with different biology and without a strong association with EBV. A study from the Austrian group also evidenced a longer FFS for EBV positive cHL [9] in univariate survival analysis, but without adjusting for covariates such as sex, age or disease stage at diagnosis. Treatment schedules are not clearly indicated in some publications, and it renders difficult the interpretation of the results in line with treatment modalities, which is one of the main drawbacks in this type of studies.

Interestingly, certain mutations identified in the LMP1 gene could reduce the antigenicity of the protein [38] and, consequently, lead to a less aggressive form of the EBV-related malignancies, but without losing its oncogenic properties. In line with the idea that viral infection could contribute to the increase in patients’ survival, certain authors supported the interpretation that LMP1-EBV could induce a more efficient activation of cytotoxic T lymphocytes (CTL) and, thus, engage a more robust antitumoral immune response [39].

In this sense, the present work presents data on the prognostic significance of EBV in a homogeneous cohort of patients treated with the same polychemotherapy (ABVD) with the aim to reduce the variability caused by this factor. Furthermore, ABVD constitutes the current standard first-line treatment as recommended in the international consensus guidelines for the management of cHL [40,41]. The results of our study are in discordance with other works evidencing a prognostic impact of EBV in cHL when adjusting for age. A study from the North Netherlands showed that the detection of EBERs impacts negatively in FFS and relative survival in cHL patients aged 50–74 years old in univariate analysis [6]. In the multivariate survival analysis, the EBV status only impacted FFS rates of cHL patients. The same negative impact was evidenced in patients above 50 years old in a study from Scotland [12]. Our work did not demonstrate a survival advantage for EBV-negative cases in the same age range (≥50 years old). In both studies, only cases of cHL were included, but other variables could explain these differences as the technique used to detect the presence of EBV, the proportion of patients with different ages within the different subgroups analyzed and, notably, variations in the treatment protocols. In the analysis from the Dutch group, six different schemas of polychemotherapy were considered, with ABVD representing only 8% of the patients included.

In agreement with previous studies, a shorter survival was evidenced in our cohort for patients presenting with advanced stages and bulky disease at diagnosis. Both parameters are part of the major prognostic scores used routinely in the clinical management of these patients [16,17].

Furthermore, the presence of EBV was not associated in the multivariate analysis with a poorer response to treatment. These results are in accordance with many studies which have consistently evidenced a lack of statistical association between EBV status and clinical outcomes in cHL [42,43,44,45]. On the one hand, one could hypothesize that EBV could enhance the immune response against the tumor as it is involved in the induction of an antigenic response [46]. Conversely, authors reporting an adverse impact of EBV expression argue a more aggressive behavior of the tumor, especially in the elderly, when functional impairment of the immune system and general ill health is expected.

The clinical course of cHL seems to be dependent on multiple factors. Although the oncogenic properties of the proteins expressed during the type II latency of EBV evidenced in HRS cells were well established, the precise mechanisms involved in the onset and progression of EBV-positive cHL are not yet fully understood. Contribution to the constitutive activation of NFKB pathway [28], the rescue of B cells with crippled mutations from apoptosis [26], promotion of cell proliferation and growth [27] and novel functions assigned to lytic proteins such as ZEBRA [29] could contribute to the progression of tumor and response to therapy in EBV-positive cHL.

Despite traditional features as the extension of disease or voluminous masses, new molecular markers need to be available at initial routine diagnosis for the early identification of high-risk patients. More intensive protocols and drugs with novel mechanisms of action could be offered to these patients. In this setting, the role of the EBV in the natural history of cHL and its prognostic role has yet to be clarified. To this end, more studies with homogeneous cohorts that avoid confounding factors will be needed in the future.

## Figures and Tables

**Table 1:** Patient demographics and outcome.

Characteristic	*n* = 88 (%)
Sex	
Male	49 (56)
Female	39 (44)
Age (years)	
Mean (range)	39 (19–82)
<30	28 (32)
≥30	60 (68)
Histological subtype	
NS	55 (63)
MC	16 (18)
LR	8 (9)
NOS	9 (10)
Ann Arbor stage	
I	2 (2)
II	36 (41)
III	10 (11)
IV	40 (46)
B symptoms at diagnosis	
Present	53 (60)
Absent	35 (40)
Bulky disease	
Present	12 (14)
Absent	76 (86)
EBV-LMP1	
Positive	36 (41)
Negative	52 (59)
IPS	
0–2	47 (53)
3–7	41 (47)
GHSG	
Limited stages	7 (8)
Intermediate stages	16 (18)
Advanced stages	65 (74)
Response to first-line therapy	
Complete remission	68 (77)
Refractory/relapsed	20 (23)

NS, Nodular sclerosis; MC, Mixed cellularity; LR, Lymphocyte-rich; NOS, Not otherwise specified; EBV-LMP1, Epstein–Barr Virus Latent Membrane Protein 1; IPS, International Prognostic Score; GSHG, German Hodgkin Study Group, Cologne, Germany.

**Table 2:** Comparison between clinical, histopathological and prognostic variables adjusted for EBV infection status.

Characteristic	EBV Positive *n* = 36 (%)	EBV Negative *n* = 52 (%)	*p*
Sex			0.084
Male	24 (67)	25 (48)	
Female	12 (23)	27 (52)	
Age (years)			**0.011**
<30	6 (17)	22 (42)	
≥30	30 (83)	30 (58)	
Histological subtype			**<0.001**
NS	13 (36)	42 (81)	
MC	15 (42)	1 (2)	
LR	5 (14)	3 (5)	
NOS	3 (8)	6 (12)	
Ann Arbor stage			0.089
I	2 (6)	0 (0)	
II	10 (28)	26 (50)	
III	5 (14)	5 (10)	
IV	19 (52)	21 (40)	
B symptoms at diagnosis			0.056
Absent	10 (28)	25 (48)	
Present	26 (72)	27 (52)	
Bulky disease			0.066
Absent	34 (95)	42 (81)	
Present	2 (5)	10 (19)	
IPS			**0.023**
0–2	14 (39)	33 (63)	
3–7	22 (61)	19 (37)	
GHSG			0.112
Limited stages	4 (11)	3 (6)	
Intermediate stages	3 (8)	13 (25)	
Advanced stages	29 (81)	36 (69)	
Response to therapy			0.925
Complete remission	28 (78)	40 (77)	
Refractory/relapsed	8 (22)	12 (23)	

NS, Nodular sclerosis; MC, Mixed cellularity; LR, Lymphocyte-rich; NOS, Not otherwise specified; EBV-LMP1, Epstein–Barr Virus Latent Membrane Protein 1; IPS, International Prognostic Score; GSHG, German Hodgkin Study Group, Cologne, Germany. Boldface font indicates statistical significance (*p* < 0.05).

**Table 3:** Univariate analysis for overall survival.

Characteristic	*N* (Mean OS *)	95% CI	*p*
Sex			0.400
Male	49 (92)	79–105	
Female	39 (82)	67–97	
Age (years)			0.103
<30	28 (80)	62–98	
≥30	60 (96)	83–109	
Histological subtype			0.137
NS	55 (80)	67–93	
MC	16 (117)	102–133	
LR	8 (86)	60–111	
NOS	9 (93)	66–121	
Ann Arbor stage			0.132
I/II	38 (87)	78–96	
III	10 (86)	60–112	
IV	40 (79)	64–94	
B symptoms at diagnosis			0.998
Present	53 (89)	77–101	
Absent	35 (87)	72–102	
Bulky disease at diagnosis			**0.009**
Present	12 (52)	37–67	
Absent	76 (93)	82–104	
EBV-LMP1			0.642
Positive	36 (93)	78–108	
Negative	52 (87)	75–99	
IPS			0.774
0–2	47 (87)	75–99	
3–7	41 (88)	73–102	
GHSG			0.282
Limited/Intermediate stages	23 (74)	66–83	
Advanced stages	65 (87)	75–99	

* OS in months. NS, Nodular sclerosis; MC, Mixed cellularity; LR, Lymphocyte-rich; NOS, Not otherwise specified; EBV-LMP1, Epstein–Barr Virus Latent Membrane Protein 1; IPS, International Prognostic Score; GSHG, German Hodgkin Study Group. Boldface font indicates statistical significance (*p* < 0.05).

**Table 4:** Univariate analysis for progression-free survival.

Characteristic	*N* (Mean PFS *)	95% CI	*p*
Sex			0.794
Male	49 (96)	81–111	
Female	39 (85)	73–98	
Age (years)			0.102
<30	28 (68)	52–83	
≥30	60 (99)	85–114	
Histological subtype			0.286
NS	55 (80)	68–93	
MC	16 (119)	105–134	
LR	8 (49)	28–70	
NOS	9 (93)	66–120	
Ann Arbor stage			**0.016**
I/II	38 (89)	82–97	
III	10 (73)	35–111	
IV	40 (76)	61–91	
B symptoms at diagnosis			0.394
Present	53 (91)	75–107	
Absent	35 (91)	72–104	
Bulky disease at diagnosis			0.055
Present	12 (47)	27–66	
Absent	76 (99)	85–112	
EBV-LMP1			0.856
Positive	36 (91)	71–111	
Negative	52 (86)	74–97	
IPS			0.214
0–2	47 (88)	77–99	
3–7	41 (89)	72–107	
GHSG			0.086
Limited/Intermediate stages	23 (75)	66–83	
Advanced stages	65 (90)	75–104	

* PFS in months. NS, Nodular sclerosis; MC, Mixed cellularity; LR, Lymphocyte-rich; NOS, Not otherwise specified; EBV-LMP1, Epstein–Barr Virus Latent Membrane Protein 1; IPS, International Prognostic Score; GSHG, German Hodgkin Study Group, Cologne, Germany. Boldface font indicates statistical significance (*p* < 0.05).

**Table 5:** Multivariate Cox regression analysis for overall survival and progression-free survival.

Characteristic (Reference)	OS *			PFS **		
	HR	95% CI	*p*	HR	95% CI	*p*
Sex (male)	**4.121**	**1.11–15.27**	**0.034**	1.379	0.45–4.20	0.571
Age (≥30 years)	2.023	0.64–6.44	0.233	1.817	0.67–4.96	0.244
Histological subtype (NS)						
MC	0.112	0.01–1.07	0.057	0.147	0.02–1.39	0.093
LR	0.866	0.14–5.46	0.878	1.427	0.27–7.64	0.678
NOS	0.189	0.03–1.22	0.080	0.292	0.06–1.47	0.134
Ann Arbor stage (IV)						
I/II	**0.074**	**0.01–0.47**	**0.006**	**0.145**	**0.04–0.57**	**0.006**
III	2.077	0.49–8.75	0.319	1.815	0.49–6.71	0.371
B symptoms (absence)	0.695	0.23–2.07	0.514	0.482	0.16–1.42	0.185
Bulky disease (presence)	**0.102**	**0.02–0.48**	**0.004**	0.289	0.08–1.06	0.062
EBV-LMP1 (positive)	0.605	0.20–1.82	0.370	0.754	0.27–2.09	0.588

* Model for OS: χ2 = 20.664; *p* = 0.024. ** Model for PFS: χ2 = 19.999; *p* = 0.029. NS, Nodular sclerosis; MC, Mixed cellularity; LR, Lymphocyte-rich; NOS, Not otherwise specified; EBV-LMP1, Epstein–Barr Virus Latent Membrane Protein 1; IPS, International Prognostic Score; GSHG, German Hodgkin Study Group, Cologne, Germany. Boldface font indicates statistical significance (*p* < 0.05).

## Data Availability

To request further information regarding the data of the study, please contact with antoniosantistebanespejo@gmail.com.

## References

[1] Weniger M.A., Küppers R. (2021). Molecular biology of Hodgkin lymphoma. Leukemia.

[2] Cartwright R.A., Watkins G. (2004). Epidemiology of Hodgkin’s disease: A review. Hematol. Oncol..

[3] Hjalgrim H., Smedby K.E., Rostgaard K., Molin D., Hamilton-Dutoit S., Chang E.T., Ralfkiaer E., Sundström C., Adami H.O., Glimelius B. (2007). Infectious mononucleosis, childhood social environment, and risk of Hodgkin lymphoma. Cancer Res..

[4] Poppema S., van Imhoff G., Torensma R., Smit J. (1985). Lymphadenopathy morphologically consistent with Hodgkin’s disease associated with Epstein-Barr virus infection. Am. J. Clin. Pathol..

[5] Jarrett R.F. (2002). Viruses and Hodgkin’s lymphoma. Ann. Oncol..

[6] Diepstra A., van Imhoff G.W., Schaapveld M., Karim-Kos H., van den Berg A., Vellenga E., Poppema S. (2009). Latent Epstein-Barr virus infection of tumor cells in classical Hodgkin’s lymphoma predicts adverse outcome in older adult patients. J. Clin. Oncol..

[7] Morente M.M., Piris M.A., Abraira V., Acevedo A., Aguilera B., Bellas C., Fraga M., Garcia-Del-Moral R., Gomez-Marcos F., Menarguez J. (1997). Adverse clinical outcome in Hodgkin’s disease is associated with loss of retinoblastoma protein expression, high Ki67 proliferation index, and absence of Epstein-Barr virus-latent membrane protein 1 expression. Blood J. Am. Soc. Hematol..

[8] Montalban C., Abraira V., Morente M., Acevedo A., Aguilera B., Bellas C., Fraga M., Del Moral R.G., Menarguez J., Oliva H. (2000). Epstein-Barr virus-latent membrane protein 1 expression has a favorable influence in the outcome of patients with Hodgkin’s Disease treated with chemotherapy. Leuk. Lymphoma.

[9] Krugmann J., Tzankov A., Gschwendtner A., Fischhofer M., Greil R., Fend F., Dirnhofer S. (2003). Longer failure-free survival interval of Epstein-Barr virus-associated classical Hodgkin’s lymphoma: A single-institution study. Mod. Pathol..

[10] Herling M., Rassidakis G.Z., Medeiros L.J., Vassilakopoulos T.P., Kliche K.O., Nadali G., Viviani S., Bonfante V., Giardini R., Chilosi M. (2003). Expression of Epstein-Barr virus latent membrane protein-1 in Hodgkin and Reed-Sternberg cells of classical Hodgkin’s lymphoma: Associations with presenting features, serum interleukin 10 levels, and clinical outcome. Clin. Cancer Res..

[11] Glavina-Durdov M., Jakic-Razumovic J., Capkun V., Murray P. (2001). Assessment of the prognostic impact of the Epstein-Barr virus-encoded latent membrane protein-1 expression in Hodgkin’s disease. Br. J. Cancer.

[12] Jarrett R.F., Stark G.L., White J., Angus B., Alexander F.E., Krajewski A.S., Freeland J., Taylor G.M., Taylor P.R.A. (2005). Impact of tumor Epstein-Barr virus status on presenting features and outcome in age-defined subgroups of patients with classic Hodgkin lymphoma: A population-based study. Blood.

[13] Huppmann A.R., Nicolae A., Slack G.W., Pittaluga S., Davies-Hill T., Ferry J.A., Harris N.L., Jaffe E.S., Hasserjian R.P. (2014). EBV may be expressed in the LP cells of nodular lymphocyte-predominant Hodgkin lymphoma (NLPHL) in both children and adults. Am. J. Surg. Pathol..

[14] Wang S., Medeiros L.J., Xu-Monette Z.Y., Zhang S., O’Malley D.P., Orazi A., Zuo Z., Bueso-Ramos C.E., Yin C.C., Liu Z. (2014). Epstein-Barr virus-positive nodular lymphocyte predominant Hodgkin lymphoma. Ann. Diagn. Pathol..

[15] Swerdlow S.H., Campo E., Pileri S.A., Harris N.L., Stein H., Siebert R., Advani R., Ghielmini M., Salles G.A., Zelenetz A.D. (2016). The 2016 revision of the World Health Organization classification of lymphoid neoplasms. Blood J. Am. Soc. Hematol..

[16] Hasenclever D., Diehl V. (1998). A prognostic score for advanced Hodgkin’s disease. International Prognostic Factors Project on Advanced Hodgkin’s Disease. N. Engl. J. Med..

[17] Engert A., Plütschow A., Eich H.T., Lohri A., Dörken B., Borchmann P., Berger B., Greil R., Willborn K.C., Wilhelm M. (2010). Reduced treatment intensity in patients with early-stage Hodgkin’s lymphoma. N. Engl. J. Med..

[18] Claviez A., Tiemann M., Peters J., Kreipe H., Schneppenheim R., Parwaresch R. (1994). The impact of EBV, proliferation rate, and Bcl-2 expression in Hodgkin’s disease in childhood. Ann. Hematol..

[19] Gulley M.L., Glaser S.L., Craig F.E., Borowitz M., Mann R.B., Shema S.J., Ambinder R.F. (2002). Guidelines for interpreting EBER in situ hybridization and LMP1 immunohistochemical tests for detecting Epstein-Barr virus in Hodgkin lymphoma. Am. J. Clin. Pathol..

[20] Kaplan E.L., Meier P. (1958). Nonparametric estimation from incomplete observations. J. Am. Stat. Assoc..

[21] Cox D.R. (1982). Regression models and life tables. J. R. Stat. Soc. Ser. B.

[22] Cheson B.D., Pfistner B., Juweid M.E., Gascoyne R.D., Specht L., Horning S.J., Coiffier B., Fisher R.I., Hagenbeek A., Zucca E. (2007). Revised Response Criteria for Malignant Lymphoma. J. Clin. Oncol..

[23] Young L.S., Dawson C.W. (2014). Epstein-Barr virus and nasopharyngeal carcinoma. Chin. J. Cancer.

[24] Brady G., MacArthur G.J., Farrell P.J. (2007). Epstein-Barr virus and Burkitt lymphoma. J. Clin. Pathol..

[25] Mancao C., Altmann M., Jungnickel B., Hammerschmidt W. (2005). Rescue of “crippled” germinal center B cells from apoptosis by Epstein-Barr virus. Blood.

[26] Dirmeier U., Neuhierl B., Kilger E., Reisbach G., Sandberg M.L., Hammerschmidt W. (2003). Latent membrane protein 1 is critical for efficient growth transformation of human B cells by epstein-barr virus. Cancer Res..

[27] Küppers R. (2003). B cells under influence: Transformation of B cells by Epstein-Barr virus. Nature reviews. Immunology.

[28] Germini D., Sall F.B., Shmakova A., Wiels J., Dokudovskaya S., Drouet E., Vassetzky Y. (2020). Oncogenic Properties of the EBV ZEBRA Protein. Cancers.

[29] Habib M., Buisson M., Lupo J., Agbalika F., Socié G., Germi R., Baccard M., Imbert-Marcille B.-M., Dantal J., Morand P. (2017). Lytic EBV infection investigated by detection of Soluble Epstein-Barr virus ZEBRA in the serum of patients with PTLD. Sci. Rep..

[30] Hong G.K., Gulley M.L., Feng W.-H., Delecluse H.-J., Holley-Guthrie E., Kenney S.C. (2005). Epstein-Barr Virus Lytic Infection Contributes to Lymphoproliferative Disease in a SCID Mouse Model. J. Virol..

[31] Rothe R., Liguori L., Villegas-Mendez A., Marques B., Grunwald D., Drouet E., Lenormand J.-L. (2010). Characterization of the Cell-penetrating Properties of the Epstein-Barr Virus ZEBRA trans-Activator. J. Biol. Chem..

[32] Connors J.M., Cozen W., Steidl C., Carbone A., Hoppe R.T., Flechtner H.-H., Bartlett N.L. (2020). Hodgkin lymphoma. Nat. Rev. Dis. Prim..

[33] Siegler G., Kremmer E., Gonnella R., Niedobitek G. (2003). Epstein-Barr virus encoded latent membrane protein 1 (LMP1) and TNF receptor associated factors (TRAF): Colocalisation of LMP1 and TRAF1 in primary EBV infection and in EBV associated Hodgkin lymphoma. Mol. Pathol..

[34] Weniger M.A., Küppers R. (2016). NF-κB deregulation in Hodgkin lymphoma. Semin. Cancer Biol..

[35] Piris M.A., Medeiros L.J., Chang K.-C. (2020). Hodgkin lymphoma: A review of pathological features and recent advances in pathogenesis. Pathology.

[36] Qi Z.-L., Han X.-Q., Hu J., Wang G.-H., Gao J.-W., Wang X., Liang D.-Y. (2012). Comparison of three methods for the detection of Epstein-Barr virus in Hodgkin’s lymphoma in paraffin-embedded tissues. Mol. Med. Rep..

[37] Jaffe E., Arber D., Harris N.L., Campo E., Quintanilla-Martinez L., Orazi A. (2016). Hematopathology.

[38] Trivedi P., Hu L.F., Chen F., Christensson B., Masucci M.G., Klein G., Winberg G. (1994). Epstein-Barr virus (EBV)-encoded membrane protein LMP1 from a nasopharyngeal carcinoma is non-immunogenic in a murine model system, in contrast to a B cell-derived homologue. Eur. J. Cancer.

[39] Landais E., Saulquin X., Houssaint E. (2005). The human T cell immune response to Epstein-Barr virus. Int. J. Dev. Biol..

[40] Eichenauer D.A., Aleman B.M.P., André M., Federico M., Hutchings M., Illidge T., Engert A., Ladetto M., ESMO Guidelines Committee (2018). Hodgkin lymphoma: ESMO Clinical Practice Guidelines for diagnosis, treatment and follow-up. Ann. Oncol..

[41] Hoppe R.T., Advani R.H., Ai W.Z., Ambinder R.F., Armand P., Bello C.M., Benitez C.M., Bierman P.J., Boughan K.M., Dabaja B. (2020). Hodgkin Lymphoma, Version 2.2020, NCCN Clinical Practice Guidelines in Oncology. J. Natl. Compr. Cancer Netw..

[42] Armstrong A.A., Lennard A., Alexander F.E., Angus B., Proctor S.J., Onions D.E., Jarrett R.F. (1994). Prognostic significance of Epstein-Barr virus association in Hodgkin’s disease. Eur. J Cancer.

[43] Enblad G., Sandvej K., Lennette E., Sundström C., Klein G., Glimelius B., Pallesen G. (1997). Lack of correlation between EBV serology and presence of EBV in the Hodgkin and Reed-Sternberg cells of patients with Hodgkin’s disease. Int. J. Cancer.

[44] Amini R.-M., Glimelius B., Gustavsson A., Ekman T., Erlanson M., Haapaniemi E., Enblad G. (2002). A population-based study of the outcome for patients with first relapse of Hodgkin’s lymphoma. Eur. J. Haematol..

[45] Herling M., Rassidakis G.Z., Vassilakopoulos T.P., Medeiros L.J., Sarris A.H., on behalf of the International Hodgkin Lymphoma Study Group (2006). Impact of LMP-1 expression on clinical outcome in age-defined subgroups of patients with classical Hodgkin lymphoma. Blood.

[46] Middeldorp J.M. (2015). Epstein-Barr Virus-Specific Humoral Immune Responses in Health and Disease. Curr. Top. Microbiol. Immunol..

